# Growth Performance and Realized Heritability in a Mass-Selected Strain of Silver Pomfret (*Pampus argenteus*)

**DOI:** 10.3390/ani15111625

**Published:** 2025-05-31

**Authors:** Chunlai Qin, Chang Li, Jie Tang, Xiang Huang, Yuanbo Li, Jiabao Hu, Yajun Wang

**Affiliations:** 1 College of Marine Sciences, Ningbo University, Ningbo 315211, China; 13282232675@163.com (C.Q.);; 2 Key Laboratory of Applied Marine Biotechnology, Ningbo University, Ministry of Education, Ningbo 315211, China; 3 Key Laboratory of Marine Biotechnology of Zhejiang Province, Ningbo University, Ningbo 315211, China

**Keywords:** *Pampus argenteus*, genetic parameters, realized heritability, artificial breeding, mass selection

## Abstract

In China, the silver pomfret (*Pampus argenteus*) is one of the most popular marine edible fish. To analyze the genetic characteristics of *P. argenteus* across three generations and evaluate whether continued progress can be made in body weight and growth traits, growth traits of the third-generation *P. argenteus* breeding and control groups were measured at 60, 90, and 120 days post-hatch. Using Pearson correlation and path analysis, we found a strong correlation between the growth characteristics of the third-generation *P. argenteus* and its body weight, with fork length and body length showing the strongest associations. Body weight, body length, and fork length all showed high levels of realized heritability at 120 days, with respective values of 0.55, 0.57, and 0.56. The genetic gain for body weight in the breeding groups over the three periods was 29.70 ± 0.58% (2.43 ± 1.17 g), which was significantly higher than the genetic gain for body length (8.90 ± 3.91%, 0.56 ± 0.29 cm) and fork length (8.08 ± 3.69%, 0.57 ± 0.33 cm). At harvest on day 120, the breeding group was 33.93% greater than the control group in terms of body weight. These results indicated that through mass selection, improvements were made to body weight of *P. argenteus*, leading to an enhancement in its growth performance. In the meantime, there was roughly equal genetic gain for body length, body weight, and fork length. These experiments showed that mass selection can effectively increase *P. argenteus* body weight, which will improve the species’ growth performance. These findings suggest that substantial genetic improvements can be achieved through mass selection for body weight, providing strong support for the ongoing *P. argenteus* selective breeding program.

## Simple Summary

The silver pomfret, a popular seafood fish in China, is crucial for marine aquaculture. To enhance its growth and farming efficiency, we studied whether selective breeding across three generations could improve its body weight and growth traits. By comparing bred and control groups at 60, 90, and 120 days after hatching, we found that body weight, body length, and fork length were strongly linked, with the highest genetic potential observed at 120 days. Selective breeding significantly boosted body weight by nearly 30% over three generations, far surpassing improvements in body length (8.9%) and fork length (8.1%). At harvest, bred fish were 34% heavier than non-bred fish. These results show that mass selection effectively increases silver pomfret body weight while maintaining balanced growth in other traits. This approach offers a practical way to improve aquaculture yields and supports ongoing efforts to breed faster-growing, higher-quality silver pomfret, benefiting both fisheries and food security.

## 1. Introduction

Mass selection is a commonly employed technique in the development of new aquaculture varieties [1]. It has been used for genetic improvement of commercially important aquatic species in the past decades [2,3]. Many economically valuable species bred through mass selection have not only increased farmers’ income but also provided people with high-quality protein [4]. For instance, Khasani and Sopian [5] obtained the larger sizes and delayed maturation stages in giant freshwater prawns (*Macrobrachium rosenbergii*) through two successive generations of mass selection. Li et al. [6] and Wang et al. [7] conducted mass selection on the shell height trait of Pacific oysters (*Crassostrea gigas*), and the shell heights of breeding lines in the first and second generations were larger than those of control lines. According to mass selection for four continuous generations, it was found by Sun et al. [8] that the average growth rate of the breeding group in Siniperca chuatsi increased by 16.3% compared with the control group. The assessment of mass selection typically involves evaluating factors such as realized heritability and genetic gains to appraise breeding effectiveness and adjust breeding programs [9,10]. Similarly, significant progress had been made in understanding genetic parameters for growth traits in fish. For example, body weight, body length, and body height traits of songpu mirror carp (*Cyprinus carpio* L.) had moderate heritability, ranging from 0.20 to 0.40, while its body width and head length displayed low heritability, with values below 0.20 [11]. Following three generations of mass selection, the realized heritability of body weight in African catfish (*Clarias gariepinus*) remained at a low level, with estimates ranging from 0.90 to 0.12 [12]. Similar results have been observed in other species, including rainbow trout (*Oncorhynchus mykiss*) [13], Nile tilapia (*Oreochromis niloticus*) [14], and Chinese tongue sole (*Cynoglossus semilaevis*) [15]. These advancements in understanding genetic parameters have supported breeding programs for various fish species. In 2020, we initiated a mass selection program targeting fast-growing lines of *P. argenteus*, highlighting its potential as a valuable marine economic fish.

Silver pomfret (*Pampus argenteus*) belongs to the *Perciformes*, *Stromateoidei*, *Stromateidae,* and *Pampus*, and is primarily distributed in the Indo-western Pacific, including regions such as the Arabian Sea, the Bay of Bengal, and the East China Sea [16,17,18]. *P. argenteus* is one of the most popular marine economic fish. *P. argenteus* is highly favored by consumers for its tender and delicious flesh, rich in high-quality protein and low fats, among other nutritional elements [19]. Moreover, *P. argenteus* with relatively heavy body weight can bring considerable economic income to farmers. Currently, the majority of *P. argenteus* products in the market are obtained through marine fishing, and their uniformity in individual size is poor. During the fishing ban period, the market is often faced with difficulties in providing freshly caught *P. argenteus* for sale. In response to this, our research team initiated mass selection breeding of *P. argenteus* in 2020. In 2021, our research team conducted mass selection of the first generation with body weight of *P. argenteus*. By 2023, we have undertaken three consecutive years of mass selection for *P. argenteus*. The realized heritability coefficient is commonly used to estimate the proportion of phenotypic variance attributed to genetic variance, with the remainder often attributed to environmental effects. It denotes the extent to which genetic factors influence the expression of the trait under specific environmental conditions [20]. To optimize the *P. argenteus* breeding program, we conducted statistical assessments of genetic parameters, including evaluations of genetic gain (for body weight from direct selection, and for other traits from indirect selection) and realized heritability. These analyses aimed to improve the accuracy and reliability of genetic evaluations and enhance the efficiency of our breeding efforts.

In this study, growth trait data from the third generation of *P. argenteus* were analyzed to estimate genetic parameters associated with body weight and to evaluate the potential for growth improvement in the selective breeding program.

## 2. Materials and Methods

### 2.1. Parental Origin

*P. argenteus* raised on the farm of “Xiangshan Bay Aquatic Offspring Seed Co., Ltd.” in Ningbo City, Zhejiang Province, China, makes up the experimental individuals. Furthermore, in China’s Fishing Area No. 209, 20,000 wild *P. argenteus* were captured in 2016. After four generations of artificial breeding, 2200 adult *P. argenteus* were randomly selected to form the fundamental group in 2022. Details of the fundamental group, breeding group, and control group, including the number of individuals, age at measurement, body weight, body length, and selection intensity, are summarized in Table 1. Mass selection for body weight was performed using a rough truncation point, selecting the top 10% heaviest individuals with a seed-retention rate of 10% and a selection intensity of 1.755 [21]. Mating was conducted using a mass spawning approach, in which multiple males and females were allowed to spawn freely in a shared environment. The mating ratio was approximately one male to two females based on the total number of males and females housed together in the spawning tanks. However, it is important to note that in a mass spawning system, the exact mating interactions are difficult to monitor, and not all males and females may have successfully participated in the mating process. This design ensured high genetic diversity and produced sufficient offspring for subsequent selection and evaluation in both the breeding and control groups. To ensure uniform growing facilities and conditions, the parent fish from both the breeding group and the control group were placed in separate circular seawater rearing tanks, with one tank for each group. The tanks had a bottom area of 26 square meters and a water level height of 1.6 m. Fertilized eggs were collected once daily for five consecutive days using a 100-mesh screen and an egg-collection device attached to the rearing tank, following spawning and spontaneous fertilization. These 5 days of collection were used to establish the offspring population. To avoid cross-contamination, the pipeline of the collection equipment was cleaned with clean saltwater after harvesting fertilized eggs from the control group, prior to gathering eggs from the breeding group. Following a filtering process to eliminate contaminants, the gathered fertilized eggs were put into two rectangular seawater rearing tanks, each measuring 8 square meters on the bottom and a water depth of 1 m. To maintain uniform raising circumstances, each instrument was used separately during the rearing procedure. The control group and breeding group were moved to circular rearing tanks with a bottom area of 26 square meters and a water-level height of 1.6 m for additional rearing once the *P. argenteus* fry reached about 5 cm in total length.

### 2.2. Hatching and Rearing

The improvements made in the first and second generations have led to improvements in the hatching and raising of *P. argenteus* fry. The Xiangshan Sea region in Zhejiang Province provided the seawater utilized for hatching and raising. It went through UV disinfection, sand filtration, and sedimentation as part of quality assurance procedures. The water was kept at a temperature between 15 and 29 °C, a pH of 7.6 and 8.2, a salinity of 26‰, and a dissolved oxygen concentration of 6 to 9 mg/L. A constant water flow was maintained following a daily water exchange of 150–250% for each raising tank. The lighting conditions were simulated throughout the day using adjustable LED lights to mimic natural light and reduce the stress brought on by exposure to uncontrolled natural light. The light intensity was regulated as follows: At 6:30, 200 lx, between 10:30 and 16:30, 50 lx, between 16:30 and 22:30, and less than 5 lx, following 22:30. *P. argenteus* were fed between 3% and 5% of their body weight during the raising phase. Refer to Table 2 for comprehensive feeding schedules.

### 2.3. Sampling and Growth Measurement

A total of 50 juvenile fish from the breeding group and 50 from the control group were randomly measured for body weight at 60, 90, and 120 days using an electronic balance with a precision of 0.01 g. The growth traits of *P. argenteus* measured in this study included total length, fork length, body length, head length, trunk length, tail length, snout length, eye diameter, caudal length, caudal height, and body height, making a total of 11 traits. These were measured using Digimizer Version 5.4.4 software. *P. argenteus* growth characteristics measurements followed the second-generation guidelines put forth by Huang et al. [22]. Of these growth traits, the mean and standard deviation were computed. In order to prevent feed from affecting weight measures, feeding was stopped 24 h before measurements.

### 2.4. Statistics on the Rate of Hatching and Fertilization

Following the parent fish’s spawning, we harvested fertilized eggs from the aquaculture pond using egg-collecting equipment that was attached to a 100-mesh screen. This includes leftover feed and parent fish excrement, as well as eggs of differing quality, such as superior and lower-quality eggs. The collected fertilized eggs were placed into a 180-L collection bucket that had been previously filled with seawater to remove any contaminants. The fertilized eggs that float on the water’s surface were collected after a 15-min settling period. We then weighed the eggs using an electronic balance to determine the total number of eggs (around 650 eggs per gram) and moved them to the hatching pool for incubation. The hatching pool and the parent fish pond were maintained at an average temperature of 21 ± 0.5 °C, ensuring a consistent environment between the two settings. After the fertilized eggs had completely differentiated into fry, the fry count was conducted. Fertilization rate and hatching rate were computed using the following formulas: fertilization rate% = (total number of fertilized eggs/total number of eggs) × 100; hatching rate% = (actual number of hatchlings/total number of fertilized eggs) × 100.

### 2.5. Growth Performance Parameters

The following formulas can be used to calculate the condition factor (CF), weight gain rate (WGR), specific growth rate (SGR), and absolute growth rate (AGR) for the breeding and control groups:

(1) $$\mathrm{CF}(g/{\mathrm{cm}}^{3})=100\times \frac{W}{L^{3}}$$

(2) $$\mathrm{WGR}(\%)=100\%\times \frac{W_{f}-W_{i}}{W_{i}}$$

(3) $$\mathrm{SGR}(\%)=100\%\times \frac{{\mathrm{lnW}}_{f}-{\mathrm{lnW}}_{i}}{D_{f}}$$

(4) $$\mathrm{AGR}=\frac{W_{f}-W_{i}}{D_{f}}$$

In Equations (1)–(4), L stands for body length (cm), W for body weight (g), W_f_ for final body weight (g), W_i_ for initial body weight (g), and D_f_ for the number of feeding days.

### 2.6. Estimation of Genetic Parameters

The genetic gain (GG) and realized heritability ($h_{R}^{2}$), for qualities strongly connected with body weight in *P. argenteus* at 60, 90, and 120 days were calculated using the following three formulas for the genetic parameter assessment. Although body length and fork length were not directly selected for in this study, their realized heritability was estimated by considering their strong correlation with body weight. The formulas are consistent with methods commonly used in aquaculture genetics studies, including those on Pacific oysters (*Crassostrea gigas*) and bay scallops (*Argopecten irradians*) [7,10].

(5) $$\mathrm{GG}=X_{S}-X_{C}$$

(6) $$\mathrm{GG}\%=100\times \frac{X_{S}-X_{C}}{X_{C}}$$

(7) $$h_{R}^{2}=R/S$$

In Equations (1)–(3), X_S_ and X_C_ denote the average size of individuals in the breeding and control groups, respectively. R is expressed as X_S_ − X_C_, and S signifies the selection differential.

### 2.7. Statistical Analyses

Genetic parameter estimates were conducted following analyses of variance homogeneity and normality to ensure the validity and reliability of the statistical models. These tests were essential to confirm that the data met the assumptions required for parametric statistical methods, such as those used in genetic parameter estimation. Statistical analysis of the morphological data was performed using Excel and SPSS 27.0, with a significance threshold of *p* < 0.05. Pearson correlation analysis was used to assess the relationships between growth traits of *P. argenteus*, identifying traits significantly correlated with body weight. Path analysis, an extension of the regression model, is a method used to examine the direct and indirect relationships between variables. In the context of this study, path analysis was applied to quantify the direct and indirect effects of various traits on body weight. This approach helps in understanding how different traits interact and contribute to the overall outcome (body weight), offering a deeper insight into the complex relationships between variables. The strength of correlation was interpreted according to Cohen’s guidelines [23], where Pearson correlation coefficients (r) less than 0.40 were considered weak, values between 0.40 and 0.59 were considered moderate, and values equal to or greater than 0.60 were considered strong. Both analyses were conducted using SPSS 27.0 software.

## 3. Results

### 3.1. The Fertilization Rate and Hatching Rate

The amounts of spawning, fertilization, and hatching in the breeding group were 65.58 × 10^4^, 27.05 × 10^4^, and 10.23 × 10^4^, respectively, while the control group had 60.40 × 10^4^, 19.72 × 10^4^, and 7.24 × 10^4^ (Table 3). In the breeding group, there was a higher rate of fertilization (41.25%) and hatching (37.82%), compared to the control group (32.69% and 32.65%), respectively.

### 3.2. Growth Performance Comparison

**Figure 1:**
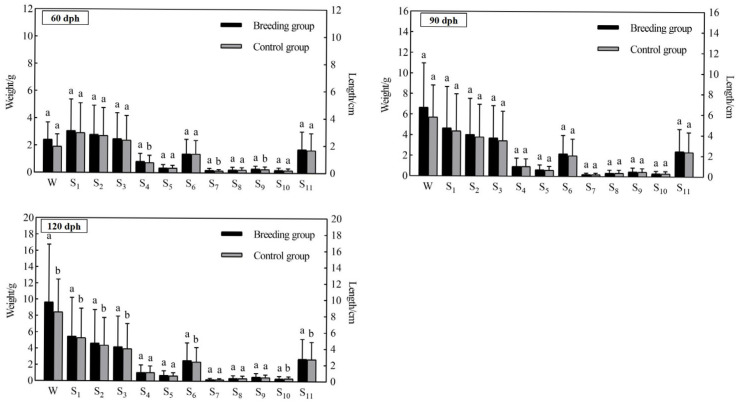
Descriptive statistics of various morphological traits of *P. argenteus* from the breeding group and control group at 60, 90, 120 days post-hatching (dph). W, body weight; S1, total length; S2, fork length; S3, body length; S4, head length; S5, trunk length; S6, tail length; S7, snout length; S8, eye diameter; S9, caudal length; S10, caudal height; S11, body height; Sample size (*n* = 50). Data are presented as mean ± SD. Different superscript letters within the same ages of *P. argenteus* indicate significant difference among means (*p* < 0.05).

Twelve growth traits of *P. argenteus* were measured at 60, 90, and 120 days (Figure 1). At 60 days, the breeding group had greater mean values of other growth characteristics, with significant differences in head, snout, and caudal lengths between the breeding and control groups (*p* < 0.05). At 90 days post-hatch, the breeding group showed a tendency toward higher mean growth trait values compared to the control group. Seven growth traits (weight, total length, fork length, body length, tail length, caudal height, and body height) showed significant differences at 120 days (*p* < 0.05). *P. argenteus* WGR during the 60–90-day period were 196.87% in the breeding group and 213.05% in the control group. The breeding group demonstrated a WGR of 52.7% in the 90–120 days, compared to 41.38% in the control group. Both the SGR and AGR were higher during the 60–90-day interval than they were throughout the 90–1200day period. Throughout the 60–120-day period, the *P. argenteus* breeding group consistently outperformed the control group in terms of WGR, SGR, and AGR (Table 4). Based on the data in Table 4, the condition factor showed no discernible variation between the breeding and control groups over the 60–120-day period.

### 3.3. Genetic Parameters

**Figure 2:**
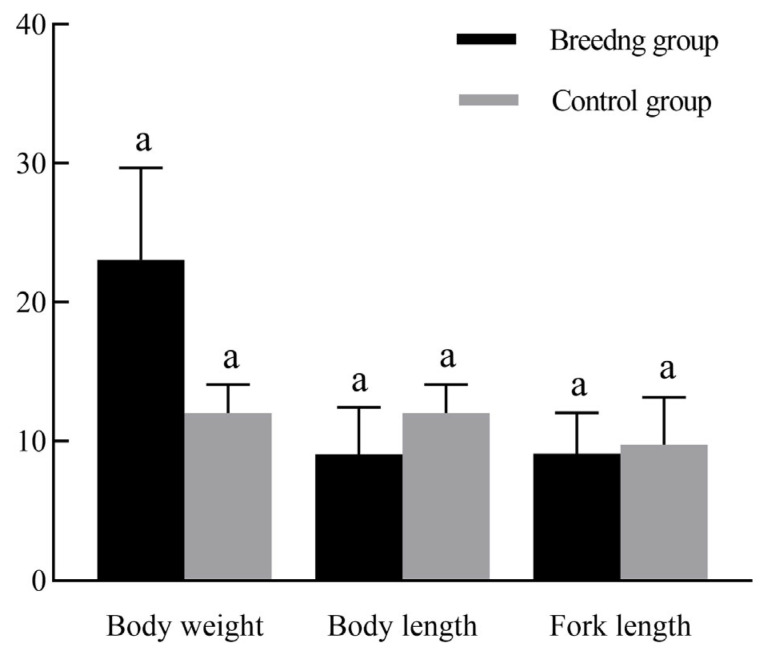
Coefficients of variation (CV%) for body weight, body length, and fork length of *P. argenteus* in breeding and control groups. Data are presented as mean ± SD. Different superscript letters within the same morphological characteristic indicate significant differences among the means (*p* < 0.05).

In this study, two growth traits—body length (the tip of the snout to the base of the caudal fin) and fork length (the tip of the snout to the fork of the tail)—were strongly linked with body weight across 11 datasets. Pearson correlation analysis was used to determine overall relationships (Supplementary Figure S1), and path analysis was conducted to quantify their direct and indirect contributions to body weight (Supplementary Table S1). At 60, 90, and 120 days, the realized heritability values were 0.49, 0.47, and 0.55, respectively, with an overall average of 0.50 ± 0.04. Correspondingly, the genetic gain percentages at these three time points were 30.90% (0.87 g), 24.14% (2.15 g), and 34.08% (4.26 g), with an average of 29.70% ± 5.08 (2.43 ± 1.17 g). The weight-related genetic gain, genetic gains percentage, and realized heritability at 120 days were greater than those at 60 and 90 days. At 60, 90, and 120 days, the genetic gain, genetic gains percentage, and realized heritability of body length showed an increasing trend. At 120 days, the values peaked at 0.91, 12.93%, and 0.57, and the averages were 0.56 ± 0.29, 8.90% ± 3.91%, and 0.44 ± 0.20, respectively. The genetic gain, genetic gains percentage, and realized heritability of fork length were similar to those of body length; they peaked at 120 days at 0.97, 12.56%, and 0.56 and averaged 0.57 ± 0.33, 8.08 ± 3.69%, and 0.40 ± 0.20, respectively (Table 5). During the three periods, the average coefficient of variation (CV) for body weight in the breeding group was 23.06%, which was much higher than the coefficients for body length and fork length. The average coefficients of variation for body weight, body length, and fork length were 23.06%, 9.11%, and 9.09%, respectively (Figure 2).

## 4. Discussion

Mass selection modifies the genetic composition within a population, aiming to shift the average phenotypic value of the target trait towards the desired direction [24]. Multiple interacting genes regulate growth parameters, and these features are also influenced by environmental factors [25]. In order to genetically improve the growth of East China Sea *P. argenteus*, mass selection was used in this study, with weight serving as the primary target attribute. In the third generation of *P. argenteus*, both Pearson correlation and path analysis confirmed the well-established association between body length, fork length, and weight. This result aligns with previous findings in other species, such as *Eleutheronema tetradactylum* [26]. According to the research, the characteristics that had the most correlation with body weight were fork length and body length. However, body length, tail length, and body height were features that substantially linked with body weight in the analysis of the first and second generations conducted by Zhang et al. [27] and Huang et al. [22]. Body length continuously showed up as the growth characteristic most significantly connected with body weight after three generations of path analysis and Pearson correlation, a pattern seen in many fish species. In a similar vein, Tong et al. [28] examined growth characteristics associated with body weight in *Paralichthys olivaceus* and found that body length had the highest relationship with body weight. The body height, tail length, fork length, and body weight of *P. argenteus* may be related genetically. However, due to variances in environmental conditions and gene effects, there are fluctuations in the connection between growth features and body weight throughout various generations [29]. Moreover, the role of environmental factors in genetic improvement cannot be overlooked. Although mass selection can effectively enhance specific growth traits, environmental conditions such as water temperature, dissolved oxygen, salinity, and light also significantly impact the growth and weight of fish [30,31]. Environmental factors, such as water temperature and dissolved oxygen, significantly influence the effectiveness of genetic improvement in fish. Moderate water temperatures and adequate dissolved oxygen levels have been shown to enhance growth performance in other species [32,33]. Future research should focus on the interactions between different growth characteristics and the impact of the environment on these characteristics. This study found the correlation between body length and body weight to be the strongest, yet other traits such as body height, tail length, and fork length also demonstrated varying degrees of correlation. Understanding the interactions between these traits can help develop more comprehensive breeding strategies aimed at multi-trait selection [34].

Genetic gain typically represents the average enhancement of a target trait in a population following a single round of mass selection [35]. More specifically, genetic gain measures how well the breeding effort improves the desired trait [36]. The per-generation genetic gain for shrimp weight ranges from 2.3% to 21.0%, with an average of 8.10% [37]. In contrast, the genetic gain for scallop body weight is between 8% and 10% [38,39]. Fish typically show greater genetic gain values for body weight compared to other growth traits, with an average genetic gain of 12.7% (relative to the trait mean of the control group) and a range of 2.3% to 42% [40]. The mandarin fish (*Siniperca chuatsi*), for example, showed an average genetic gain of 12.14% in body weight per generation, with a cumulative genetic gain range of 6.94% to 17.25% over four generations. These values reflect significant progress achieved after four generations of continuous selective breeding, which also produced successful offspring [8]. This study found that the third generation of *P. argenteus* achieved a genetic gain of 29.70 ± 5.08% relative to the trait mean and 2.43 ± 1.17 g on the observed scale in body weight, resulting from one round of selection in the third generation. *P. argenteus* had shown effective breeding results and acquired considerable genetic gain values after three generations of continuous mass selection. Over the course of the three periods, the genetic gains in body weight were substantially greater than those in body length and fork length. This suggests that mass selection enhanced the frequency of beneficial genes in the population, resulting in offspring with superior growth traits [41]. Additionally, the impacts of the selection plan on the rise of body weight were particularly noticeable in our breeding and selection program, demonstrating the viability of our selection technique. The similar CF values suggest that while significant differences in growth traits (WGR, SGR, and AGR) were observed between the breeding and control groups, the breeding program did not adversely affect the overall condition or proportional robustness of the fish [42]. In this study, we observed that the WGR of the breeding group was lower than that of the control group during the 60 to 90 days, showing a certain degree of growth lag at this stage. However, after 90 days, the growth advantage of the breeding group began to emerge. This early growth difference may reflect variations in individual developmental rhythms or adjustments in energy allocation strategies. Specifically, the selected individuals may have allocated more energy to organ development and tissue remodeling during the early stage, rather than directly increasing body weight, which was consistent with the energy-utilization characteristics observed in the early developmental stages of zebrafish [43]. This stability in CF supports the efficiency and sustainability of the breeding strategy used in this study.

In this study, a population-based natural mating approach was employed, enabling selected individuals to freely pair and reproduce within the same environment. Prior to spawning, the gonads of the *P. argenteus* were treated to synchronize gonadal development, ensuring they reached the optimal physiological state for reproduction. This intervention effectively improved the success rate of natural mating [44]. However, it is acknowledged that there may be discrepancies between the selected individuals and those that actually participate in reproduction [45]. Not all selected individuals necessarily engage in the breeding process, which can affect the selection intensity, also known as the selection differential. In a population-based natural mating system, the randomness of mating makes it difficult to track which selected individuals successfully reproduce and pass their genetic material to the next generation [46]. Consequently, selection differentials cannot be updated or corrected, which may bias the estimation of realized heritability for the target traits. To address this, future research should explore more precise methods for monitoring reproductive contributions. For instance, genetic markers could be used to clearly identify the individuals contributing to the offspring, or controlled breeding designs could track the reproductive success of each selected individual [47,48]. Accurately assessing realized heritability is essential for understanding genetic progress and optimizing breeding strategies. Improving the precision of heritability estimates in these breeding programs will provide a stronger theoretical foundation for future genetic improvements.

We conducted mass selection for body weight on the third generation of *P. argenteus*, assessing the realized heritability of body weight and the correlated response of traits linked to body weight, such as body length and fork length. The realized heritability at 120 days was 0.55 for body weight, 0.57 for body length, and 0.56 for fork length. Realized heritability, according to Fan [49], could be divided into three categories: higher than 0.4 was regarded as high, between 0.2 and 0.4 were intermediate, and lower than 0.2 was indicative of low realized heritability. The realized heritability of *P. argenteus* body weight was estimated to be greater than 0.4, indicating a notable genetic influence on individual body weight. However, environmental factors still play a significant role in body weight variation [50,51]. The realized heritability of body weight varies across low, medium, and high levels in different fish species. For instance, in three generations of mass selection (G0, G1, G2, and G3), Sun et al. [52] evaluated the genetic parameters of body weight in turbot (*Scophthalmus maximus*, Linnaeus), and their findings revealed a relatively high realized heritability in the G2 generation, whereas in the other generations, it ranged from moderate to low. The realized heritability of body weight in *Cyprinus carpio* var. *Quanzhouensis* was assessed by Xu et al. [53], who found that body weight has a high heritability (0.45, *p* < 0.01). Realized heritability is employed to infer the potential selection response of specific traits in the population [54]. Traits with stronger additive genetic effects typically exhibit higher realized heritability, as these effects directly influence the phenotype, and heritability reflects the proportion of phenotypic variation attributable to genetic factors [7]. This results in more effective selection. In aquaculture breeding, the CV is closely related to genetic potential, as traits with greater CV often exhibit higher levels of variability and respond better to selective breeding. Thus, traits with greater CV are typically prioritized as target breeding traits [55,56]. In this study, the CV was analyzed for three traits in the breeding group of *P. argenteus*: body weight (CV: 23.06%), body length (CV: 9.11%), and fork length (CV: 9.09%). Among these, body weight had the highest CV. Consequently, the breeding group shows prospective benefits in the mass selection of *P. argenteus*, indicating its importance in genetic improvement [57,58]. According to Liu et al. [59,60], the huge yellow croaker (*Pseudosciaena crocea*) has a larger CV in its body weight than in its body length and other growth traits. This is consistent with our findings, which show that in *P. argenteus*, body weight had a larger CV than body length and other phenotypic traits. However, external environmental factors, such as water quality, feeding regimes, and variability in rearing conditions, can significantly affect growth performance, including body weight, as they may influence the fish’s metabolism, nutrient absorption, and overall health. As a result, direct selection based solely on weight may lead to restricted breeding effectiveness [61]. Implementing indirect selection based on body length and fork length during the third generation could effectively reduce the potential for environmental factors to interfere with breeding outcomes. Because body length, fork length, and body weight are positively correlated during *P. argenteus* growth, selection based on these traits may facilitate indirect selection for body weight, even though traits with a lower CV do not necessarily have a lower environmental impact. A preliminary version of this study was previously published as a preprint [62].

## 5. Conclusions

The findings indicate that significant genetic gains in body weight of *P. argenteus* can be achieved through mass selection. At day 120, the genetic gain in body weight for the breeding group was 4.26 g, representing a 34.08% increase relative to the trait mean. This suggests the effective improvement of body weight traits through selective breeding, with the breeding group showing a notable increase in body weight compared to the control group. These findings provide valuable technical assistance and a scientific foundation for the ongoing refinement of *P. argenteus* breeding programs, potentially serving as a guide for breeding other economically important species.

## Figures and Tables

**Table 1:** Parental data for the third generation of *P. argenteus* breeding program.

Group	N (Animals)	Age (Days)	Body Weight (g) (Mean ± SD)	Body Length (mm) (Mean ± SD)	Sires	Dams	Selection Intensity
Fundamental	2200	270	52.42 ± 11.51	--	--	--	--
Breeding	200	270	54.26 ± 10.45	124.73 ± 7.73	67	133	1.755
Control	200	270	47.94 ± 9.68	122.29 ± 9.68	71	129	--

**Table 2:** Feeding sequence and feeding amount of *P. argenteus* seedlings.

Birth Day	Body Length of the Fish/(mm)	Feed Types	Feeding Amount	Feeding Time
Start feeding–5 days	˂5 mm	-	-	6:20 a.m. 9:30 a.m. 11:00 a.m. 1:30 p.m. 3:00 p.m. 5:00 p.m. 8:00 p.m. 10:20 p.m.
5–10	5–8 mm	Rotifer	1 individual/mL	
11–15	8–12 mm	Rotifer	2–3 individuals/mL	
16–20	12–18 mm	Rotifer	2–3 individuals/ml	
		Artemia nauplii	0.5–1 individuals/mL	
21–26	18–25 mm	Artemia nauplii	1–2 individuals/mL	
27–34	20–30 mm	Artemia nauplii	1–2 individuals/mL	
		2# YuBao compound feed	-	
35–55	25–46 mm	2# YuBao compound feed	-	
56–67	29–50 mm	2# YuBao compound feed	-	
		3# YuBao compound feed	-	
68–90	33–66 mm	3# YuBao compound feed	-	
91–120	>33 mm	3# YuBao compound feed	-	
		SaiFeng Nian compound feed	-	
>120	>40 mm	SaiFeng Nian compound feed	-	

2#, 3# YuBao compound feeds were purchased from Hayashikane Sangyo Co., Ltd. (Shimonoseki City, Japan), Feed Business Division Chofu Plant, Japan. Saifeng Nian cooperates with the purchase of feed from Ningbo Tianbang Feed Technology Co., Ltd. (Ningbo, China).

**Table 3:** Summary of the egg-laying information of *P. argenteus* from the breeding group and control group.

Group	The Number of Eggs Laid (×10^4^)	The Number of Fertilized Eggs (×10^4^)	The Number of Hatched Fishes (×10^4^)	Fertilization Rate	Hatching Rate
Breeding group	65.58	27.05	10.23	41.25	37.82
Control group	60.40	19.72	7.24	32.65	36.69

**Table 4:** Weight gain rate (WGR), specific growth rate (SGR), absolute growth rate (AGR) and condition factor (CF) of breeding group and control group at different ages.

	WGR (%)		SGR (%)		AGR (g/Feeding Day)		CF (g/cm^3^)	
	Breeding Group	Control Group	Breeding Group	Control Group	Breeding Group	Control Group	Breeding Group	Control Group
60–90 dph	196.87	213.05	3.63	3.80	24.23	20.03	4.30	3.80
90–120 dph	52.70	41.38	1.41	1.15	19.26	12.18	3.48	3.53
60–120 dph	353.33	342.59	5.04	4.96	43.49	32.21	3.32	3.58

**Table 5:** Standardized genetic gains (GG), genetic gains percentage (GG%), realized heritability ($h_{R}^{2}$) of *P. argenteus* from the breeding group.

Traits	Age	GG	GG%	$h_{R}^{2}$
Body weight	Day 60	0.87 g	30.90	0.49
	Day 90	2.15 g	24.14	0.47
	Day 120	4.26 g	34.08	0.55
Mean		2.43 ± 1.17 g	29.70 ± 5.08	0.50 ± 0.04
Body length	Day 60	0.21 cm	5.11	0.21
	Day 90	0.55 cm	8.68	0.53
	Day 120	0.91 cm	12.93	0.57
Mean		0.56 ± 0.29 cm	8.90 ± 3.91	0.44 ± 0.20
Fork length	Day 60	0.17 cm	3.52	0.14
	Day 90	0.56 cm	8.16	0.50
	Day 120	0.97 cm	12.56	0.56
Mean		0.57 ± 0.33 cm	8.08 ± 3.69	0.40 ± 0.20

## Data Availability

The original contributions presented in this study are included in the article/Supplementary Materials. Further inquiries can be directed to the corresponding authors.

## Supplementary Materials

The following supporting information can be downloaded at: https://www.mdpi.com/article/10.3390/ani15111625/s1, Figure S1: Correlation analysis of morphometric traits in *Pampus argenteus*. Heatmaps show Pearson correlation coefficients among various morphometric traits at 60 days (a), 90 days (b), and 120 days (c) post-hatch. Darker colors indicate stronger correlations. The hierarchical clustering dendrograms reveal trait groupings based on similarity in correlation structure. Table S1. Direct and indirect effects of morphometric traits on body weight of *P. argenteus.*
