# Supplementary material for: Growth Performance and Realized Heritability in a Mass-Selected Strain of Silver Pomfret (Pampus argenteus)
Source: Animals (Basel). 2025 May 31;15(11):1625. doi: 10.3390/ani15111625 (PMC12153585; doi:10.3390/ani15111625)
Supplement: Supplementary file 1 [file animals-15-01625-s001.zip › animals-3572209-supplementary.pdf]

Supple Figure S1: Correlation analysis of morphometric traits in *Pampus argenteus*. Heatmaps show Pearson correlation coefficients among various morphometric traits at 60 days (a), 90 days (b), and 120 days (c) post-hatch. Darker colors indicate stronger correlations. The hierarchical clustering dendrograms reveal trait groupings based on similarity in correlation structure.

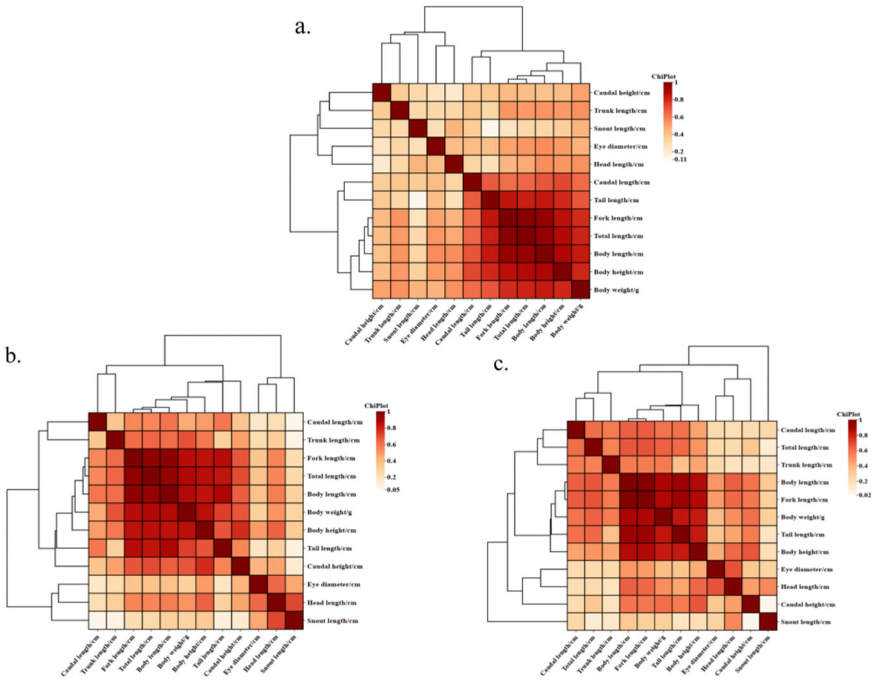

Supple Table S1. Direct and indirect effects of morphometric traits on body weight of *P. argenteus*

Dph	Traits	Correlation coefficient	Path coefficient	Indirect path coefficient			Total
				Fork Length	Body Length	Total Length	
60	Fork length	0.789^{**}	0.284	-	0.691	0.382	1.073
	Body length	0.830^{**}	0.727	-0.270	-	0.373	0.096
	Total length	0.807^{**}	0.392	-0.277	0.691	-	0.414
90	Fork length	0.858^{**}	-0.077	-	0.460	0.475	0.935
	Body length	0.871^{**}	0.477	-0.074	-	0.467	0.393
	Total length	0.873^{**}	0.495	-0.074	0.450	-	0.376

	Fork length	0.877^{**}	-0.052		0.874	0.055	0.929
120	Body length	0.888^{**}	0.885	-0.051	-	0.054	0.003
	Total length	0.634^{**}	0.081	-0.035	0.586	-	0.551
